# Clinical characteristics, treatment and prognosis of children with unilateral retinoblastoma and intracranial segment of Retrobulbar optic nerve invasion

**DOI:** 10.1186/s12886-020-01768-4

**Published:** 2021-01-14

**Authors:** Yi-Zhuo Wang, Yi Zhang, Dong-Sheng Huang, Ji-Tong Shi, Jian-Min Ma, Bin Li, Xiao-Lin Xu, Yan Zhou, Hua-Li Gu

**Affiliations:** 1 Department of Pediatrics, Beijing Tongren Hospital, Capital Medical University, No. 2, Xihuan South Road, Yizhuang Economic and Technological Development Zone, Beijing, 100176 China; 2 Department of Ophthalmology Oncology, Beijing Tongren Hospital, Capital Medical University, Beijing, China; 3 Department of Ophthalmology Science, Beijing Tongren Hospital, Capital Medical University, Beijing, China

**Keywords:** Retinoblastoma, Optic nerve invasion, Intracranial segment of retrobulbar optic nerve, Prognosis

## Abstract

**Background:**

To analyze the clinical characteristics, treatment and prognosis of children with unilateral retinoblastoma (RB) and intracranial segment of retrobulbar optic nerve invasion.

**Methods:**

A total of 14 children with unilateral RB and intracranial segment of retrobulbar optic nerve invasion were enrolled in this retrospective study from January 2009 to December 2018. Clinical characteristics, treatment and prognosis were collected and analyzed. Survival curves were calculated by Kaplan-Meier method.

**Results:**

Of 14 cases, there were 7 male and 7 female, ranging in age from 22.85 to 121.97 months (median, 41.03 months). Seventy-one percent of patients came from first-tier cities in China and effected in the left eye. Magnetic resonance imaging (MRI) results indicated that all patients presented with thickened and enhanced optic nerve and intracranial segment of optic nerve invasion. Nine patients received comprehensive therapeutic regimen (chemotherapy, eye enucleation, radiotherapy and intrathecal therapy). The patients were followed up to December 2019, with a median follow-up of 20.6 months. The median disease specific survival was 48.99 ± 8.62 months, and the overall survival (OS) rate was 64.3%. Radiotherapy and comprehensive therapeutic regimen had significant impact on survival time (all *p* < 0.05).

**Conclusions:**

The overall prognosis of unilateral RB patients with intracranial segment of retrobulbar optic nerve invasion was poor. Chemotherapy and surgical treatment were necessary, but more attention should be paid to radiotherapy and intrathecal therapy for improving prognosis.

## Background

Retinoblastoma (RB) is the most common primary intraocular malignancy in children, but a rare paediatric cancer with a reported incidence worldwide of 1:15,000 to 1:20,000 live births [1]. RB is unilateral in about 60% of cases with a median age at diagnosis of two years [2]. Leukocoria is the most common initial presenting sign of RB which is seen in 60 to 80% of newly diagnosed patients, followed by strabismus [3, 4]. Diagnosis of RB is made by an examination of the fundus under general anesthesia. Computed tomography (CT) scans, magnetic resonance imaging (MRI) and ultrasound are used to confirm the diagnosis and assess the local extension [5]. Prognosis and survival depends on early diagnosis and appropriate treatment.

China is the most populous country in the world and documents about 1000 new cases of RB every year, accounting for 1/8 of all cases worldwide [6]. Good medical conditions and modern technology have increased overall survival rate of RB to exceed 95% in developed countries [7]. However, in developing countries, patients with RB are mostly diagnosed at advanced stages [8]. Patients with advanced RB usually have poor prognosis, especially those with an intracranial segment of optic nerve invasion [9]. There is no special clinical manifestation in this kind of patients, but orbital MRI show that thickened and enhanced optic nerve invaded the intracranial segment [10]. In addition, the report of RB with intracranial segment of optic nerve invasion is extremely rare. Our hospital collects nearly 3000 cases of RB clinical data from January 2009 to December 2018, and screen out only 14 cases of unilateral RB patients with intracranial segment of retrobulbar optic nerve invasion. In this study, we aimed to retrospectively analyze the clinical characteristics, treatment and prognosis of children with unilateral RB and intracranial segment of retrobulbar optic nerve invasion.

## Materials and methods

### Patients

We retrospectively reviewed 14 children with unilateral RB and intracranial segment of retrobulbar optic nerve invasion from January 2009 to December 2018. The inclusion criteria were as follows: 1) Patients diagnosed with unilateral RB for the first time in our hospital; 2) patients with orbital magnetic resonance imaging (MRI) before chemotherapy suggesting invasion of the intracranial segment of retrobulbar optic nerve; 3) patients with age < 14 years; 4) patients undergoing chemotherapy, radiotherapy, surgery, intrathecal therapy and follow-up according to comprehensive treatment of our hospital. The exclusion criteria were as follows: 1) patients with trilateral RB; 2) patients with recurrent invasion of the intracranial segment of the optic nerve after treatment; 3) patients with distant metastasis of bone, bone marrow and lymph nodes at the time of diagnosis; 4) patients with severe congenital deficiency, including immune deficiency, liver and kidney patients, etc.; 5) patients with a family history of mental illness; 6) patients with a family history of RB. This study was approved by the ethics committee of the Beijing Tongren Hospital, Capital Medical University (number, TRECKY2019–034). Written informed consent was obtained from all participants and their guardians.

### Diagnostic criteria

Orbital CT scan, orbital MRI, eye ultrasound and fundus examinations were performed in all patients. In addition, neuron specific enolase (NSE) in serum and cerebrospinal fluid (CSF) of patients were analyzed using the electrochemiluminescence with the NSE Kit (Roche, US). Lactate dehydrogenase (LDH), a non-specific tumor marker, was detected by chemical immunoassay. The pathology examination of CSF was performed by hematoxylin eosin staining. In our hospital, all patients whose imaging or serology test showed RB were also undergoing bone marrow aspiration. If bone marrow involvement was confirmed, re-examination should be conducted every 3 months until complete remission of bone marrow.

Diagnosis of retinoblastoma was usually clear from presenting signs and clinical examination [10]. MRI was used to assess invasion of the optic nerve. All tumors were classified (stage 0-IV) according to the International Retinoblastoma Staging System (IRSS) proposed by Chantada et al. [11].

### Therapy

Patients received chemotherapy, eye enucleation, radiotherapy or intrathecal therapy. Pre-surgical chemotherapy was sustained for 4–6 cycles, and 21 days were one cycle of chemotherapy. Multi-drug combination chemotherapy was adopted in our therapy, including vincristine (age < 3 years, 0.05 mg/kg; age ≥ 3 years, 1.5 mg/m^2^; day 1), carboplatin (age < 3 years, 18.6 mg/kg; age ≥ 3 years, 560 mg/m^2^; day 1), etoposide (age < 3 years, 5 mg/kg; age ≥ 3 years, 150 mg/m^2^; day 1–2). Post-surgical chemotherapy was sustained for 3–4 cycles. Radiotherapy was performed for children over 3 years old after post-surgical chemotherapy, and the radiotherapy dose of orbital was 36–45 Gy. Chemotherapy was continued after radiotherapy, and the total chemotherapy cycle was 16–18.

In each cycle of the chemotherapy, lumbar puncture and intrathecal injection were performed for each patient with methotrexate (MTX), cytosine arabinoside (Ara-c), and dexamethasone (Dex) according to the following regimen: age < 12 months: MTX (5.0 mg), Ara-c (12.0 mg), Dex (2.0 mg); age 12–24 months: MTX (7.5 mg), Ara-c (15.0 mg), Dex (2.0 mg); age 2–3 years: MTX (10.0 mg), Ara-c (25.0 mg), Dex (5.0 mg); age 3–12 years: MTX (12.5 mg), Ara-c (35.0 mg), Dex (5.0 mg); age > 12 years: MTX (12.5 mg), Ara-c (35.0 mg), Dex (5.0 mg).

### Clinical assessment and follow-up

During the treatment period, routine CSF cytology examination was performed before each cycle of the chemotherapy, and fundus examinations and orbital MRI were performed every 4–8 weeks. Follow up every 3 months in the first 1–2 years, every 6 months in the 3–5 years, and once a year from the 6th year after treatment. During follow-up, we monitored patients for tumor recurrence though fundus examinations and orbital MRI. The prognosis of patients was indicated by the overall survival (OS). OS was calculated from the day of first admission to hospital to the time of the last follow-up or death.

### Statistical analysis

All statistical analyses were performed by using SPSS version 22.0 (SPSS Institute. IL.USA). Quantitative data were expressed as means±standard deviations (SD). Qualitative data were expressed as number and percentage and were compared using Fisher’s exact test. Survival curves were calculated by Kaplan-Meier method. Statistical significance was set at *p* < 0.05.

## Results

### Baseline characteristics

**Fig. 1:**
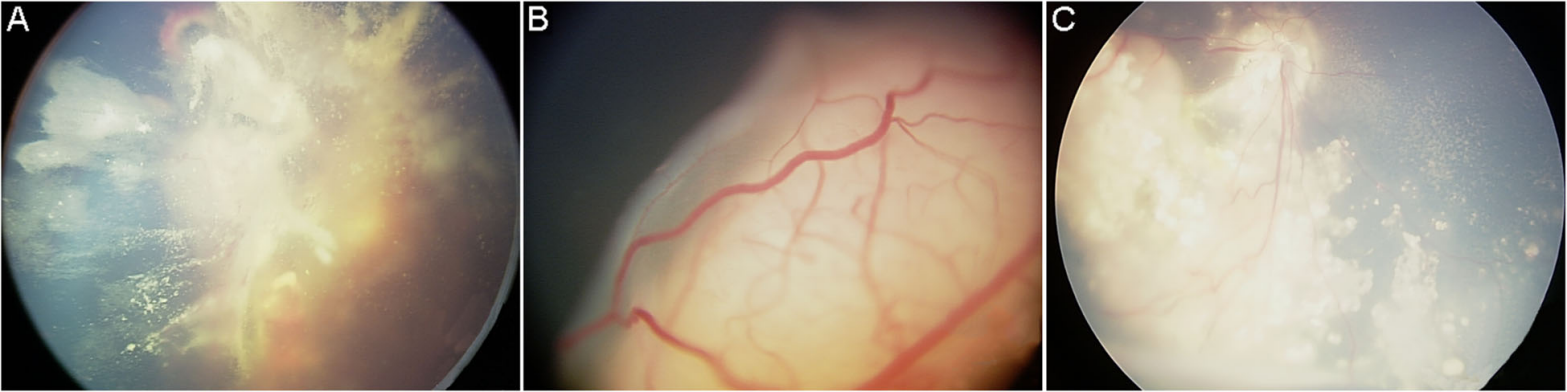
Representative images of fundus examinations. **a**, Left fundus of one 3-year-old girl showed a large tumor space occupying with hemorrhage under the retina, wildly vitreous cavity seeding, and partial detachment of retina. **b**, Left fundus of one 3-year-old boy showed a large tumor space occupying and obvious vascular network under the retina, and partial detachment of retina. **c**, Right fundus of one 10-year-old girl showed tumor cell invasion under the retina, wildly vitreous cavity seeding, and calcification

A total of 14 children (7 males, 7 females; median age: 41.03 months; range from 22.85 to 121.97 months) with unilateral RB and intracranial segment of retrobulbar optic nerve invasion were enrolled in this retrospective study from January 2009 to December 2018. The baseline characteristics of included patients were showed in Tables 1 and 2. The majority of cases were diagnosed after the age of 3 years (78.6%). All patients were found to be at stage IV. Leukocoria was the most common presenting sign in the diagnosis early RB, accounting for 42.9% (6/14). The median time from first symptom to diagnosis was 3.79 months, with most patients (11/14, 78.6%) diagnosed more than four weeks after first symptom. Bone marrow examinations were negative in all patients. Most patients (10/14, 71.4%) came from first-tier cities in China. Seventy-one percent of the tumors were in the left eye, and 28.6% were in the right eye **(**Fig. 1**)**.

**Table 1:** Baseline characteristics of included patients

Characteristic	Number (n, %)
Age (years), < 3/≥3	3 (21.4)/11 (78.6)
Gender, M/F	7 (50.0)/7 (50.0)
Eye affected, left/right	10 (71.4)/4 (28.6)
City, first-tier/second-tier	10 (71.4)/4 (28.6)
Presenting signs	
Leukocoria	6 (42.9)
Strabismus	3 (21.4)
Others	5 (35.7)
Time from first symptom to diagnosis (mo), median	3.79 (0.1–17.35)
< 4 weeks	3 (21.4)
≥ 4 weeks	11 (78.6)

*M* male, *F* female, *mo* months

**Table 2:** Clinical characteristics of all patients

No.	Gender	Age (years)	Eye affected	RB staging (IRSS)	Serum NSE (ng/dl)	NSE in CSF (ng/dl)	CSF cytology	serum LDH (U/L)	Total chemotherapy cycle	Radiotherapy	Radiotherapy dose (Gy)	Eye enucleation	Outcome	Time from first symptom to diagnosis (mo)	Cause of death
1	Male	2.24	Left	IV	31.6	12.6	N	352.0	15.00	No	–	Yes	Death	0.46	Meningeal metastasis
2	Female	10.16	Right	IV	20.2	5.3	N	216.0	8.00	Yes	45.00	Yes	Survival	2.07	–
3	Male	2.50	Right	IV	35.6	12.3	N	290.0	12.00	No	–	Yes	Death	7.95	Meningeal metastasis
4	Female	3.44	Left	IV	23.1	12.7	N	312.0	12.00	Yes	39.6	Yes	Survival	0.30	–
5	Male	4.81	Right	IV	20.8	17.2	N	250.0	14.00	No	–	Yes	Survival	1.87	–
6	Male	3.40	Left	IV	26.3	1.25	N	297.0	7.00	Yes	48.80	Yes	Survival	17.35	–
7	Male	6.30	Left	IV	65.0	294.7	P	311.0	6.00	Yes	45.00	Yes	Death	0.1	Meningeal metastasis
8	Female	3.60	Left	IV	24.80	8.4	N	124.0	10.00	No	–	No	Death	15.44	Meningeal metastasis
9	Male	3.28	Left	IV	15.7	8.4	N	359.0	14.00	Yes	43.20	Yes	Survival	2.56	–
10	Female	4.44	Left	IV	27.50	89.3	P	224.0	19.00	Yes	43.20	Yes	Survival	11.66	–
11	Female	1.90	Left	IV	21.0	6.5	N	256.0	9.00	Yes	39.60	Yes	Survival	6.47	–
12	Female	3.07	Left	IV	21.0	8.5	N	2240.0	15.00	Yes	43.20	Yes	Survival	5.19	–
13	Female	6.85	Right	IV	34.9	25.1	N	264.0	15.00	Yes	43.20	Yes	Survival	2.46	–
14	Male	3.00	Left	IV	18.5	10.2	N	234.0	6.00	No	–	No	Death	5.03	Meningeal metastasis

*No* number, *mo* month, *NSE* neuron specific enolase, *CSF* cerebrospinal fluid, *LDH* lactate dehydrogenase, *N* negative, *P* positive (small round malignant cells)

### Clinical characteristics

**Fig. 2:**
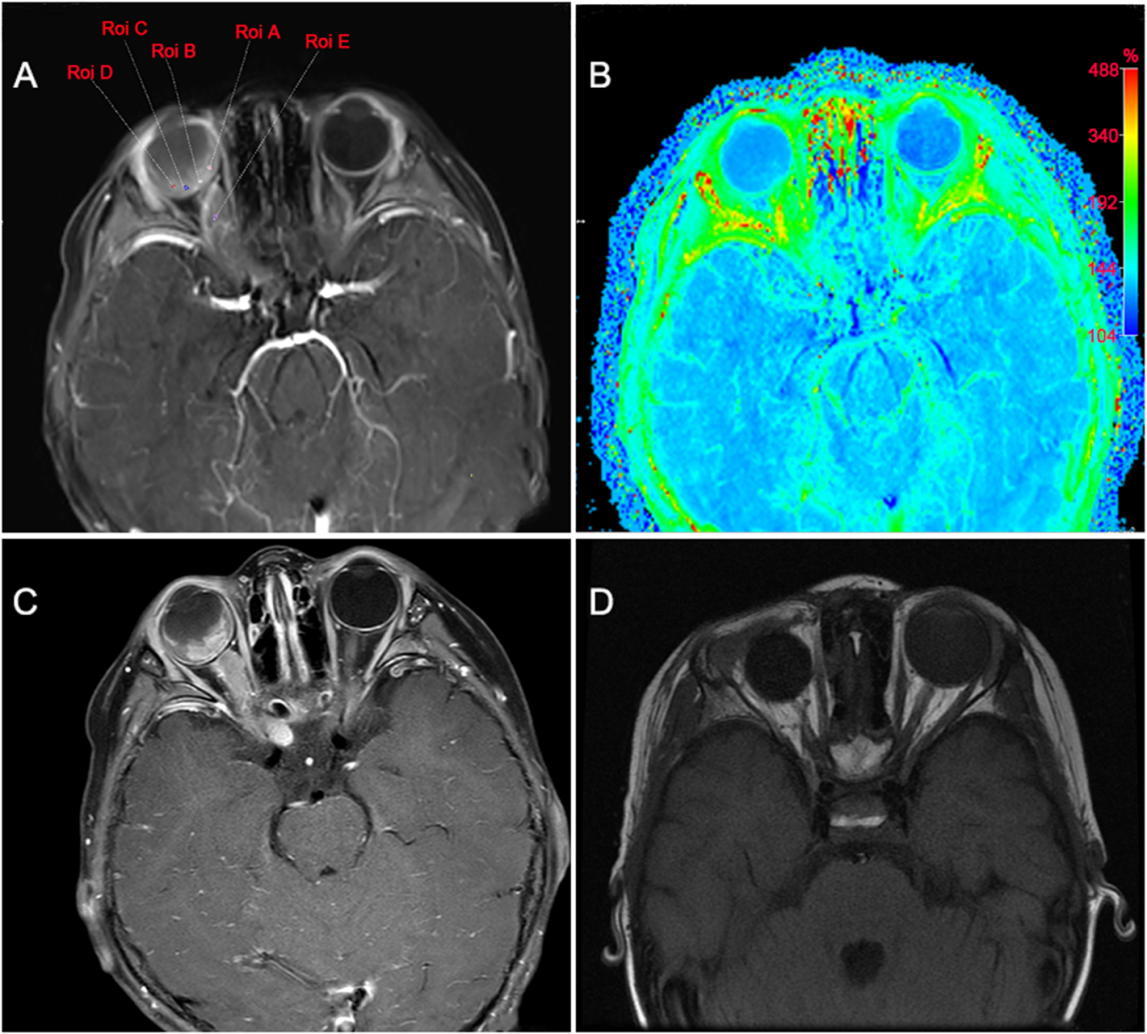
Representative images of orbital MRI in one unilateral RB patients with intracranial segment of retrobulbar optic nerve invasion. **a**, Orbital MRI in one patient showed a space-occupying lesion in the left eyeball, thickened optic nerve and intracranial segment invasion. **b**, Magnetic resonance angiography; **c**, Orbital MRI showed enhancement in inintraorbital and intracanalicular segments of optic nerve during treatment; **d**, MRI image of left eye after enucleation and chemotherapy. MRI, magnetic resonance imaging; RB, retinoblastoma

**Fig. 3:**
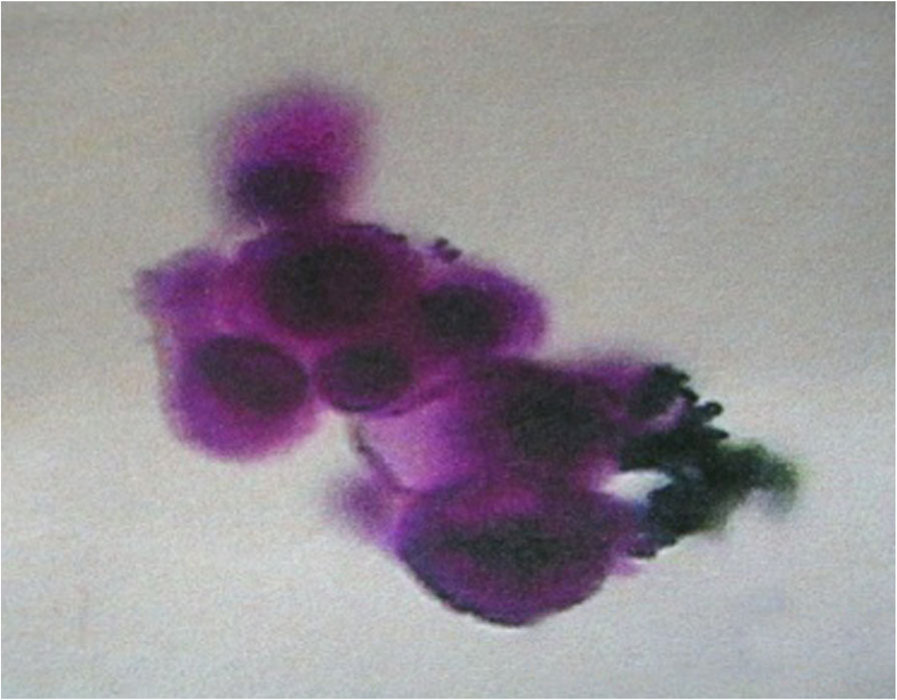
Representative image of CSF cytology in unilateral RB patients with intracranial segment of retrobulbar optic nerve invasion. Malignant small round cell as shown in the CSF smear (× 400); CFS, cerebrospinal fluid; RB, retinoblastoma

Orbital MRI in all patients showed abnormal intraocular signal, thickened and enhanced optic nerve invaded the intraorbital, intracanalicular and intracranial segment. In addition, the optic nerve extends from the eyeball to the intracranial, so the intracranial optic nerve invasion must involve the intraorbital and intracanalicular invasion. Moreover, tumor cell invasion was found in the cut end of optic nerve of all patients after eye enucleation. In addition, 8 patients had choroidal invasion, 3 patients had scleral invasion, 2 patients had scleral invasion. Representative orbital MRI results of unilateral RB patients with intracranial segment of retrobulbar optic nerve invasion were shown in Fig. 2. As shown in Table 2, all patients had serum NSE values above the normal reference range (0–15.2 ng/dl) before treatment, with a median serum NSE value of 23.95 ng/dl (range, 15.7–65.0 ng/dl). The median value of NSE in CSF was 11.25 ng/dl (range, 5.3–294.7 ng/dl), of which 6 cases were higher than the normal reference range (0–10.1 ng/dl). The pathology of CSF in 2 cases with significantly elevated NSE indicated small round malignant cells **(**Fig. 3**)**. In addition, the median serum LDH value was 409.5 U/L (range, 124–2244 U/L), of which 5 cases were higher than the normal reference range (0–245 U/L).

### Treatment and prognosis

Among the 14 patients, 2 (14.3%) were administered with chemotherapy combined with intrathecal therapy, 3 (21.4%) with chemotherapy, eye enucleation and intrathecal therapy, 9 (64.3%) with chemotherapy, eye enucleation, radiotherapy and intrathecal therapy **(**Table 2**)**.

**Fig. 4:**
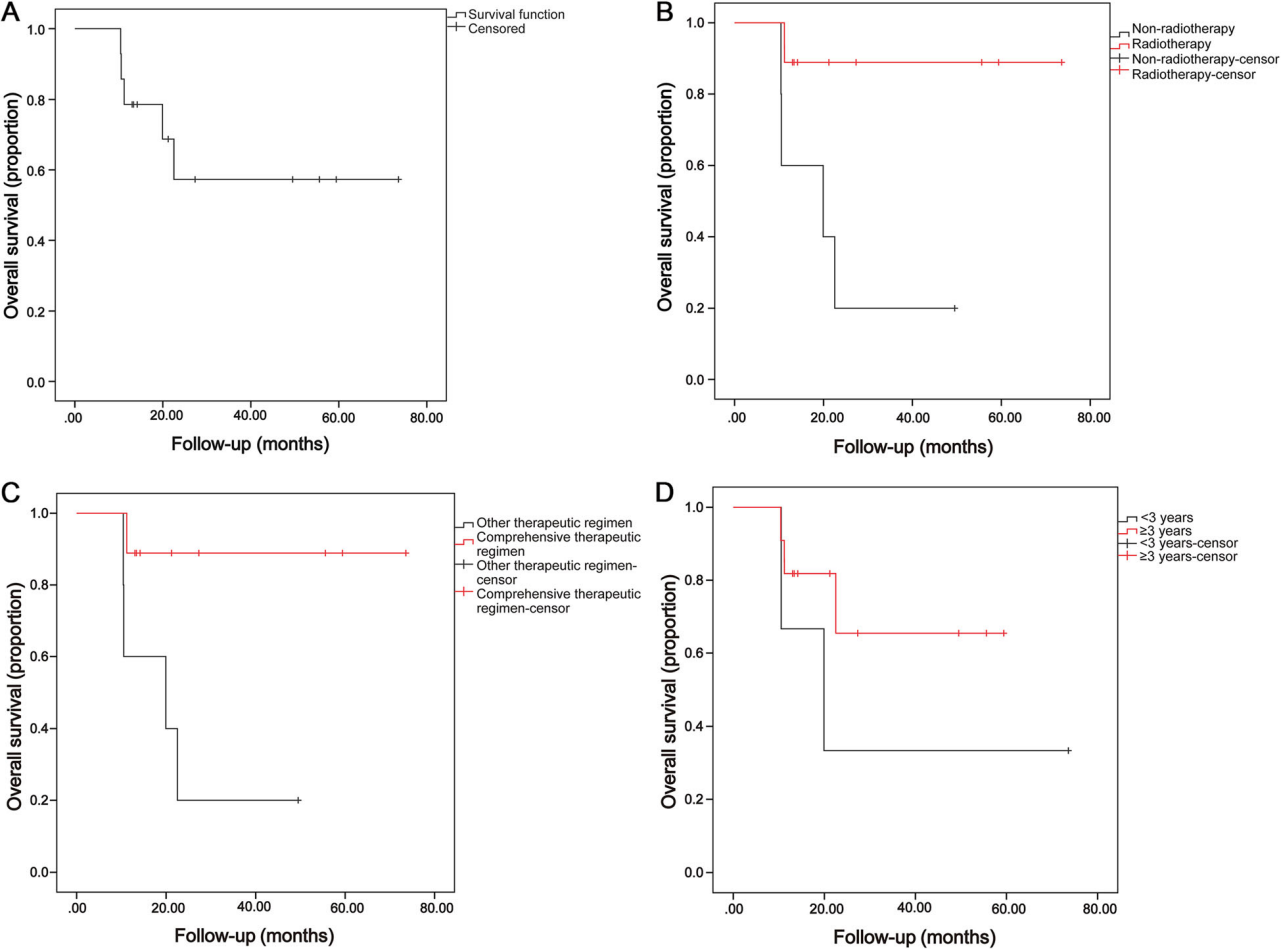
Overall survival according to different categories. **a**, Overall survival of all 14 patients; **b**, Kaplan-Meier survival curves according to radiotherapy (radiotherapy and non-radiotherapy); **c**, Kaplan-Meier survival curves according to therapeutic regimen (comprehensive therapeutic regimen and other therapeutic regimen); **d**, Kaplan-Meier survival curves according to age group (age < 3 and ≥ 3 years). Comprehensive therapeutic regimen, chemotherapy, radiotherapy, eye enucleation and intrathecal therapy

The patients were followed up to December 2019, with a median follow-up of 20.6 months (range, 10.4–73.58 months). At the study closing date, there were 9 patients alive, 5 patients had died due to meningeal metastasis. The mean disease specific survival was 48.99 ± 8.62 months, and the OS rate was 64.3% **(**Fig. 4a**)**.

Radiotherapy was associated with a longer disease specific survival (OS, 66.7 ± 6.5 months) compared with non-radiotherapy (OS, 22.6 ± 6.4 months, *p* = 0.023) **(**Fig. 4b**)**. Considering the therapeutic regimen **(**Fig. 4c**)**, the OS of patients received comprehensive therapeutic regimen (chemotherapy, radiotherapy, surgery and intrathecal injection) was longer than that of patients received other therapeutic regimen (66.7 ± 6.5 vs. 22.6 ± 6.4 months, *p* = 0.047). No statistically significant differences in OS were identified when the study group was divided by age (age < 3 and ≥ 3 years, 34.7 ± 16.0 vs. 44.5 ± 7.2 months, *p* = 0.281, Fig. 4d).

## Discussion

RB is the most common intraocular malignancy in childhood, which seriously endangers children’s vision and life [12]. Optic nerve invasion has been found to be correlated with worse survival rate in RB patients [13]. In addition, patients with unilateral RB and intracranial segment of optic nerve invasion are extremely rare. In this study, we aimed to retrospectively analyze the clinical characteristics, treatment and prognosis of children with unilateral RB and intracranial segment of retrobulbar optic nerve invasion.

Diagnosis of RB was usually clear from clinical examination and presenting signs [14]. The most common sign was leukocoria and strabismus [10]. In this study, we found that 42.9% of patients presented with leukocoria, and 21.4% with strabismus. Based on a large data from USA, the mean age at diagnosis of RB was 18 months, with unilateral cases diagnosing at a mean age of 24 months and bilateral diagnosing at 12 months [15]. In a study of 1457 cases of RB from Indian, the mean age at diagnosis was 29 months, with unilateral cases diagnosing at a mean age of 34 months and bilateral at 21 months [16]. Compared with developed countries, the diagnosis of RB in developing countries was usually delayed [17]. In this study, we found that the mean age at diagnosis was 22.85 months in unilateral cases, which consistent with the reports from USA. The possible reason was that most patients (71.4%) came from first-tier cities (Beijing, Tianjin, Shanghai, Chongqing, etc) in China. These cities were more medical-developed than other regions in the Chinese Mainland.

Treatment strategies for RB involved chemotherapy, radiotherapy and surgical enucleation [18]. Sullivan et al. performed a prospective, graduated intensity approach for adjuvant therapy based on pathologic findings of primarily enucleated eyes in patients with unilateral RB [19]. They found that patients in the high risk groups (pathology showed sclera invasion and/or tumor at the cut end of the optic nerve) were successfully treated with no evidence of recurrence after only six courses (each course of chemotherapy was 21 days) of adjuvant chemotherapy. Since the children in this group had been diagnosed with the intracranial segment of optic nerve invasion, the treatment methods were mainly systemic chemotherapy, radiotherapy, eye enucleation, and intrathecal therapy in each cycle of the chemotherapy. A total of 9 patients received comprehensive therapeutic regimen (chemotherapy, eye enucleation, radiotherapy and intrathecal therapy). After 20 months of follow-up, the OS rate improved to 64.3%, which was higher than that reported by Gunduz et al. [20]. They found that all patients with distant and CNS metastasis were deceased during a mean follow-up of 24 months (range, 4–62 months). In addition, we also found that radiotherapy and comprehensive therapeutic regimen were associated with a longer disease specific survival. At the study closing date, there were 9 patients (all received comprehensive therapeutic regimen) alive, 5 patients (4 did not receive radiotherapy and 2 did not receive eye enucleation) had died due to meningeal metastasis. These results indicated that patients with intracranial segment of optic nerve invasion should be treated comprehensively to obtain better clinical effect.

## Conclusions

The overall prognosis of unilateral RB patients with intracranial segment of retrobulbar optic nerve invasion was poor. Chemotherapy and surgical treatment were necessary, but more attention should be paid to radiotherapy and intrathecal injection for improving prognosis.

## Data Availability

The datasets used and/or analysed during the current study are available from the corresponding author on reasonable request.

## Abbreviations

RB: Retinoblastoma
MRI: Magnetic resonance imaging
OS: Overall survival
CT: Computed tomography
CSF: Cerebrospinal fluid
ICRB: International Classification of Retinoblastoma
NSE: Neuron specific enolase
LDH: Lactate dehydrogenase
MTX: Patient with methotrexate
Ara-c: Cytosine arabinoside
Dex: Dexamethasone
SD: Means±standard deviations
